# Targeting the Tumor Microenvironment in Triple-Negative Breast Cancer: Emerging Roles of Monoclonal Antibodies and Immune Modulation

**DOI:** 10.3390/cancers18030412

**Published:** 2026-01-28

**Authors:** Stephanie Figueroa, Niradiz Reyes, Raj K. Tiwari, Jan Geliebter

**Affiliations:** 1 School of Arts and Sciences, Cornell University, Ithaca, NY 14853, USA; sf563@cornell.edu; 2 Genetics and Molecular Biology Research Group, Basic Sciences Department, School of Medicine, University of Cartagena, Cartagena 130014, Colombia; nreyesr@unicartagena.edu.co; 3 Department of Pathology, Microbiology and Immunology, New York Medical College, Valhalla, NY 10595, USA; raj_tiwari@nymc.edu; 4 Department of Otolaryngology, New York Medical College, Valhalla, NY 10595, USA

**Keywords:** triple-negative breast cancer (TNBC), tumor microenvironment (TME), monoclonal antibodies (mAbs)

## Abstract

Triple-negative breast cancer (TNBC) is an aggressive and clinically challenging subtype of breast cancer characterized by the absence of estrogen receptor, progesterone receptor, and HER2 expression. This molecular phenotype narrows the availability of targeted therapies and contributes to high rates of early relapse, therapeutic resistance, and poor clinical outcomes. Mounting evidence pinpoints the tumor microenvironment (TME) as a central driver of TNBC progression, immune evasion, and resistance to treatment. The TME encompasses a complex and dynamic network of immune and stromal cells, extracellular matrix components, and soluble mediators that collectively shape tumor behavior and influence therapeutic response. Notably, TNBC often displays an immunologically active microenvironment, marked by high levels of tumor-infiltrating lymphocytes and immune checkpoint expression, opening a window for immune-based therapeutic strategies. This narrative review summarizes current knowledge on the cellular, molecular, and structural features of the TNBC tumor microenvironment, with particular focus on immunosuppressive mechanisms mediated by tumor-associated macrophages, myeloid-derived suppressor cells, cancer-associated fibroblasts, and dysfunctional T cells. We describe the clinical development and therapeutic impact of monoclonal antibodies, including immune checkpoint inhibitors and antibody–drug conjugates. Additionally, we discuss strategies aimed at modulating the TME to enhance monoclonal antibody efficacy, including immune cell reprogramming, extracellular matrix remodeling, cytokine/chemokine blockade, and combination treatment strategies. Finally, we highlight the role of biomarker-driven patient stratification and personalized therapeutic strategies, addressing current challenges and future directions in TME-targeted drug development. Together, these insights underscore the potential of integrating immune modulation and monoclonal antibody-based therapies to improve outcomes for TNBC patients.

## Simple Summary

Triple-negative breast cancer (TNBC) is a subtype of breast cancer that lacks expression of estrogen, progesterone, and HER2 receptors. This cancer represents a major therapeutic challenge due to limited targeted treatment options and an immunosuppressive tumor microenvironment (TME). Recent advances highlight the potential of monoclonal antibodies (mAbs), especially those targeting immune checkpoints and TME components, as promising therapeutic agents. This narrative review highlights current knowledge on the interaction between TNBC and its microenvironment, emphasizing how mAbs disrupt immunosuppressive signaling, modulate stromal architecture, and enhance immune effector functions. We further explore combination strategies and ongoing clinical developments aimed at optimizing mAb efficacy within the complex TME of TNBC.

## 1. Introduction

The absence of estrogen receptor (ER), progesterone receptor (PR), and human epidermal growth factor receptor 2 (HER2) expression is the landmark of triple-negative breast cancer (TNBC), reflecting both its molecular identity and its clinical behavior [1]. This subtype accounts for approximately 15–20% of all breast cancers and is characterized by noticeable biological heterogeneity, high histologic grade, and aggressive clinical features [2]. TNBC is more frequently diagnosed in younger patients and is associated with an increased risk of early recurrence, visceral metastasis, and reduced overall survival compared with hormone receptor-positive and HER2-positive breast cancers [3]. The characteristic phenotype of TNBC excludes this subtype from the therapeutic benefit of endocrine and HER2-targeted therapies that have significantly improved outcomes in other breast cancer subtypes. For this reason, systemic chemotherapy with anthracyclines and taxanes, administered as neoadjuvant or adjuvant therapy, remains the main management approach for both, early and advanced stages with the primary goals of reducing tumor size and eradicating micrometastasis [4,5]. Although a subset of patients achieve a favorable initial response, including pathological complete response following neoadjuvant treatment, many experience rapid disease recurrence and the development of chemoresistant tumors [6].

The dependence on non-specific cytotoxic therapy stresses the biological aggressiveness of TNBC and emphasizes the pressing need for alternative treatment approaches that surpass conventional chemotherapy to achieve more robust clinical benefit. Consequently, TNBC represents a major unmet clinical need, emphasizing the importance of identifying novel therapeutic strategies and prognostic biomarkers capable of improving risk stratification and treatment outcomes in the affected patient population. Abundant research studies have shown that the tumor microenvironment (TME) has an essential role in TNBC progression [7], therapeutic resistance [8], and immune evasion [9]. In the TME, cancer cells engage in many complex interactions with both stromal cells and tumor-infiltrating immune cells; this is performed either through direct contact or by the secretion of soluble factors, with the subsequent changes in the phenotypes of stromal and immune cellular components [10]. Many cytokines, chemokines, enzymes, growth factors, inflammatory molecules, and matrix remodeling enzymes can be identified as the soluble factors that sustain the progression of TNBC [11,12]. Therefore, a better understanding of the cellular and molecular components of TNBC and their interactions in the TME is essential for unraveling the mechanisms behind tumor cell immune evasion strategies [11].

The limited scope of conventional chemotherapy in TNBC have fueled the interest in therapeutic strategies that move beyond direct cytotoxicity and instead exploit tumor-specific vulnerabilities [4,13,14]. In this context, monoclonal antibodies (mAbs), and other immunotherapeutic approaches that targets the TME, have emerged in the last few years as promising alternatives against both tumor-intrinsic and immune-mediated mechanisms used for cancer to progress [11,15,16]. Through the selective targeting of immune checkpoints, tumor-associated antigens, stromal components, and/or soluble mediators within the TME, mAbs bear the potential to enhance antitumor immune responses while minimizing off-target toxicity [17,18]. Moreover, modulation of the TME can disrupt immunosuppressive signaling networks, restore effective immune surveillance, and improve sensitivity to existing therapies. The integration of mAb-based therapies with strategies designed at reprogramming the TME components, such as CAFs, or modulation of TAM polarization, represents rational and evolving strategies for overcoming therapeutic resistance and improving clinical outcomes in patients with TNBC [19]. This review aims to provide an up-to-date overview of the evolving landscape of TME-targeted therapies in TNBC, with a specific focus on monoclonal antibodies and strategies that modulate immune responses. We discuss the biological foundations of TME components relevant to TNBC, current clinical developments, and future directions for combining immune modulation with precision-targeted therapies to improve patient outcomes.

## 2. The Tumor Microenvironment in TNBC

**Figure 1:**
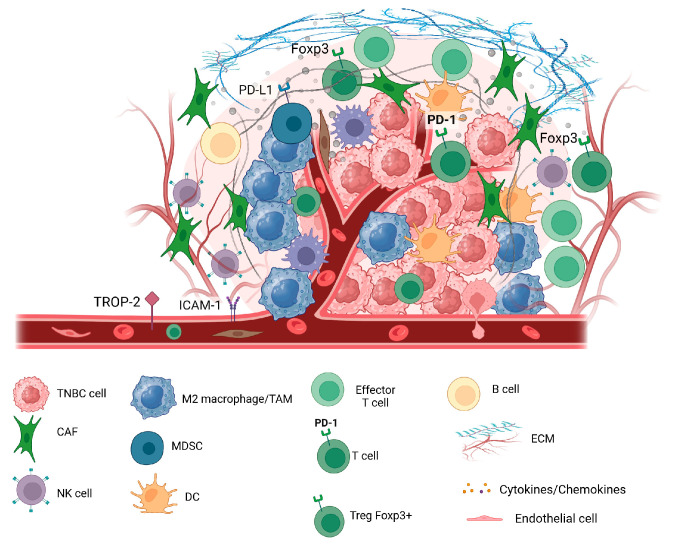
The Tumor Microenvironment in Triple-Negative Breast Cancer. Schematic representation of the tumor microenvironment in triple-negative breast cancer. Interactions between tumor cells, immune populations, stromal elements, and extracellular matrix components contribute to immune suppression, therapeutic resistance, and disease progression. Created in https://BioRender.com.

During the carcinogenesis process, tumor cells interact with the extracellular elements creating a unique environment called TME (Figure 1). TME is composed of 10–20% of cancer cells and around 80% of other cells, both immune and non-immune cells, and signaling factors, mostly cytokines and chemokines [20]. Similar to other solid tumors, in addition to cancerous cells, the TME in TNBC consist of a complex network of stromal cells (including endothelial cells, pericytes, fibroblasts, adipocytes and mesenchymal stem cells), immune cells (including T cells, B cells, plasma cells, NK cells, macrophages, myelogenous suppressor cells, dendritic cells and neutrophils), extracellular matrix components, and signaling molecules, that collectively impact tumor behavior and response to therapy [21,22,23]. The TME of solid tumors have immune cells at varied proportions, ranging from subtle infiltration to overt inflammation. This inflammatory infiltrate is composed of several cell types that participate in both innate and adaptive immunity, and displaying diverse functions.

While a pro-tumoral role has been recognized for the cells of the innate immune system, the B and T cells, belonging to the adaptive immune system, mainly show anti-tumor potential [24]. Unlike all the other BC subtypes, TNBC often shows an immunologically “hot” microenvironment, with elevated levels of tumor-infiltrating lymphocytes (TILs) and immune checkpoint expression, making it a feasible target for immunotherapeutic strategies [25,26]. Among these, monoclonal antibodies (mAbs) targeting immune checkpoints [27], stromal components [28], or tumor-associated antigens [29], have emerged as promising tools to reprogram the TME and enhance anti-tumor immunity. For example, mAbs directed against tumor-associated antigens may engage the immune system to attack the cancer cells through antibody-dependent cellular cytotoxicity (ADCC), and this mechanism in turn can lead to local increases in inflammatory cytokines and chemokines, ultimately driving an inflammatory repolarization of the tumor microenvironment [30,31].

### 2.1. TME Cellular Components and Their Role in TNBC

#### 2.1.1. Tumor-Associated Macrophages (TAMs)

TAMs are the most prevalent innate immune cells in the TME of TNBC, making over 50% of the tumor mass, and mainly exhibiting an M2 phenotype that favors an immunosuppressive environment that helps the tumor evade the immune system [32,33]. M2-TAMs also induce the formation of new blood vessels that support tumor growth, facilitate tumor cell invasion and metastasis [33]. High levels of infiltrating TAMs correlate with reduced expression of the epithelial marker E-cadherin, elevated expression of the mesenchymal marker fibronectin, higher cancer aggressiveness, and poorer prognosis in TNBC [34].

#### 2.1.2. Myeloid-Derived Suppressor Cells (MDSCs)

MDSCs are a type of innate immature myeloid cells produced from abnormal myelopoiesis and recruited to the TME where they promote tumor cell survival, angiogenesis, invasion and metastasis [35]. Tumor cells produce chemokines that regulate the recruitment process, with CCL2 and CCL5 as the main chemokines implicated in MDSC migration to tumors [36,37]. These immune cells have been shown to favor postoperative recurrence and premetastatic niche formation [38]. They behave as potent immunosuppressor cells frequently observed in the TME of many tumor types, including TNBC, where they have been linked to poor outcomes in these patients [39].

#### 2.1.3. Cancer-Associated Fibroblasts (CAFs)

CAFs are activated fibroblasts making up the major stromal component in the various types of malignancies [40]. This cell type plays a significant role in tumorigenesis, favoring growth and invasion of tumor cells by sustaining a TME that is unique for each cancer type [41,42]. Cellular interactions between CAFs and tumor cells contribute to modify the surrounding extracellular matrix (ECM) components and the basement membrane, favoring cancer progression [41]. They play a role in the metabolic and immune reprogramming of the tumor microenvironment causing an impact not only in tumor growth and metastasis, but also on adaptive resistance to chemotherapy, which is linked to poor outcomes [40]. Elevated CAF levels in TNBC are associated with tumor aggressiveness, recurrence, and poor outcomes [43].

In TNBC, the actions of CAFs on tumor cells contribute to tumor growth and progression in two main ways: indirectly by promoting immune evasion, and directly by activating signaling pathways within tumor cells, leading to disruption of normal cellular functions, such as cell cycle regulation and programmed cell death [44]. CAFs indirectly promote tumor progression by shaping an immunosuppressive microenvironment. Besides their ability to recruit immune cells that favor tumor growth, CAFs have been shown to also affect the function of several immune cells inducing them to acquire an immunosuppressive phenotype by multiple mechanisms, including secretion of different cytokines and chemokines, and mutual interactions that mediate the recruitment and function of innate and adaptive immune cells [45]. Moreover, the marked heterogeneity of CAFs, both in origin and function, suggests that immune evasion in cancer, including TNBC, may be mediated by distinct CAF subpopulations with specialized immunomodulatory roles [41,46].

Regarding direct contribution to tumor growth and progression, emerging in vitro evidence indicates that CAFs remodel the extracellular matrix into a stiff, fibrotic scaffold that generates mechanical stress and activates mechanotransduction pathways such as YAP/TAZ, by this means accelerating G1/S cell cycle transition in cancer cells [47]. In parallel, accumulating evidence implicate CAFs in cancer initiation and progression through the regulation of apoptosis via multiple mechanisms, including the exosomal transfer of CAF-derived miRNAs that suppress apoptotic signaling and the secretion of interleukin-6, which promotes apoptosis resistance through the upregulation of anti-apoptotic Bcl-2 family proteins [48].

#### 2.1.4. Tumor Infiltrating Lymphocytes (TILs)

TILs represent a diverse population of immune cells, mainly T cells, that infiltrate the TME following chemokine gradients and inflammatory signals that mediate the recruitment process from peripheral blood to tumor sites [49]; they have a significant role in the immune response against cancer, either as proinflammatory or immunosuppressive, depending on their abundance, cellular composition, functional status, and specific interactions within the tumor microenvironment [49,50]. These cells are classified into several different subtypes, mainly CD3+ T cells followed by CD20+ B cells. CD3+ T cells include CD8+ cytotoxic T lymphocytes (CD8+ TILs), CD4+ helper T lymphocytes, and Foxp3+ regulatory T lymphocytes (Foxp3+ Tregs) [12]. Depending on their location within the tumor, two specific subpopulations of TILs are recognized: stromal TILs (sTILs) that make up the majority of TILs and locate in the stromal region directly adjacent to the tumor, and the intratumoral TILs (iTILs) found within the tumor itself and make up a smaller fraction [51].

The cellular composition of TILs has predictive and prognostic value in breast cancer, especially in Triple-Negative (TNBC) and HER2-positive subtypes, where high TILs levels correlate with better overall survival and disease-free survival [52,53,54]. Metastases are often characterized by a lower number of TILs compared to primary tumors and patients with low TILs in metastatic tumors had a significantly lower overall survival than those with intermediate TILs level [55]. TIL-based immunotherapy represents a promising approach that exploits the inherent specificity and potential of the adaptive immune system to fight cancer, making them a powerful tool for personalized anticancer treatments [49].

### 2.2. Molecular and Structural Features

#### 2.2.1. Hypoxia, Extracellular Matrix (ECM) Remodeling

The extracellular matrix (ECM) is the highly dynamic and complex non-cellular component surrounding cells within all tissues and organs, that provides essential physical scaffolding for the cellular constituents and necessary biochemical and biomechanical signals required for tissue morphogenesis, differentiation and homeostasis [56]. ECM contains around 300 different proteins with important roles in tissue development, function, and homeostasis [57]. It is the major non-cellular component of the TME and has received increasing attention for its significant involvement in breast cancer progression, metastasis, and therapy resistance [58,59].

Solid tumors are commonly affected by hypoxia as oxygen availability is limited due to rapid tumor growth and impaired blood supply due to a disordered vasculature. In normal cells, hypoxia is associated with cell cycle arrest and apoptosis, but tumor cells are able to adapt and survive hypoxic conditions, allowing for tumor growth, invasion, and metastasis [60,61]. The best studied mechanism used by tumors to adapt to hypoxia involves hypoxia-inducible factors (HIFs), with several studies showing an overactive HIF-1 pathway in TNBC, compared to other subtypes, as more than 80% of TNBC patients overexpressing HIF-1 [61,62]. The important role of hypoxia and the extracellular matrix (ECM) in altering cell metabolism and tumor metastasis has been recently recognized [61]. Although these two features were initially thought to independently influence cell metabolism and metastasis, several lines of evidence points out to mechanisms involving a crosstalk between the ECM and hypoxia in the TME, as important forces impacting on breast cancer progression [61].

#### 2.2.2. Cytokine and Chemokine Signaling in TNBC

Mutual interactions among malignant and non-malignant cells (including stromal and inflammatory cells) favor activation of cellular processes mediated by different growth factors, chemokines, and cytokines, ultimately leading to extracellular matrix remodeling, cell migration, neo-angiogenesis, invasion, drug resistance, and immune evasion [63]. Evidence from in vitro and in vivo research have shown that CCL5, IL-6, and IL-8, along with angiogenic factors, play an important role in TNBC tumor growth and metastasis via crosstalk between cancer cells and stromal components [64,65].

## 3. Role of the TME in Tumor Progression and Resistance to Therapy

### 3.1. The TME as a Driver of Therapeutic Resistance in TNBC

Mounting evidence have uncovered the essential role of the TME components in the regulation of tumor initiation, disease progression, metastatic development, and more significantly, the therapeutic response, which is the main factor affecting the outcomes of TNBC patients receiving anticancer treatments [66]. Dynamic interactions taking place between malignant cells and surrounding stromal, immune, and extracellular matrix components actively shape tumor behavior by promoting immune evasion, sustaining chronic inflammation, and facilitating adaptive resistance to treatment [67,68]. Immunosuppressive cell populations, aberrant cytokine and chemokine signaling, hypoxic conditions, and extracellular matrix remodeling collectively create a protective niche that limits drug penetration and attenuates antitumor immune activity [69,70,71]. These microenvironment-driven mechanisms enable tumor cells to survive cytotoxic and immune-based therapies, contributing to disease persistence and relapse [72]. Understanding how the TME influences treatment response is therefore essential for the development of effective therapeutic strategies aimed at overcoming resistance and improving long-term outcomes in patients with TNBC.

### 3.2. T-Cell Exclusion and Dysfunction

Although T cell-based cancer therapies are promising strategies intended to induce complete responses in patients against several types of cancer, a significant proportion of patients who initially responded to T cell-based therapies do not achieve durable clinical responses [73]. Accumulating evidence shows that stromal cells of the TME mediate this restriction by excluding T cells from the area surrounding cancer cells, establishing an immune-privileged microenvironment [74]. Mechanisms responsible for resistance or short-term response to these therapies remain largely unknown, with some studies suggesting that efficacy of immunotherapy is restricted by the generation of dysfunctional T cells in the TME [73]. Intratumoral T cells shows a wide range of dysfunctional states, characterized by a loss of effector functions and complex suppressive signals that arise within the tumor microenvironment, with the upregulation of programmed cell death-1 (PD-1) on T cells recognized as a major marker of T cell dysfunction [75]. Currently, new promising therapies for TNBC patients are under development, especially immune checkpoint inhibitors that regulate T-cell function, such as antibodies against programmed cell death 1 (PD-1) or its ligand PD-L1. However, only a few patients exhibit complete or partial response to anti-PD-1 treatment, with dysfunction of CD8+ T cells identified as one of the key reasons for the immune escape of TNBC [76].

## 4. Monoclonal Antibodies as Therapeutic Agents in TNBC

### 4.1. Monoclonal Antibody-Based Therapies

Monoclonal antibodies (mAbs) have become important therapeutic agents against cancer, used alongside or in combination with surgery, radiation, and chemotherapy. They may target antigens specific to or overexpressed by tumor cells leading to tumor cell death by diverse mechanisms, with blockade of growth factor receptor signaling recognized as the main direct mechanism of action [77,78]. Indirect mechanisms of action of mAbs include complement-dependent cytotoxicity (CDC), antibody-dependent cellular phagocytosis (ADCP), and antibody-dependent cellular cytotoxicity (ADCC), which require the engagement of components of the host immune system [17,77]. Thanks to their versatility, mAbs have been used in cancer therapy in several ways, such as antibody–drug conjugates (ADC), targeting of pro-tumorigenic compounds in the TME, bispecific T cell engagers (BiTEs), and immune checkpoint inhibitors [77,79]. As TNBC lack expression of ER, PR and HER2, the use of monoclonal antibodies alone or in combination to other therapeutic options, such as chemotherapy, radiotherapy, and targeted therapies, has become potential treatment options for this difficult to treat cancer [80] (Table 1).

### 4.2. Immune Checkpoint Inhibitors

Immune checkpoint inhibitors are recognized as novel and promising tools for cancer immunotherapy [81]. Humanized monoclonal antibodies that target immune checkpoint proteins such as PD-1 and CTLA-4 inhibitory receptors on T cells, and their ligands on some cancer cells, such as PD-L1, have been employed with success to treat patients with different types of cancer [82]. Between 2004 and 2023 a total of 331 immunotherapy-based trials were registered in breast cancer, enrolling 48,844 patients, with 252 trials (76.1%) designed to include the TNBC subtype [83]. Analysis of the outcomes of clinical trials, showed that among 90 trials that reported results, 47 (52.2%) were positive, while 43 (47.8%) were negative, representing a low return on the investment in immunotherapy trials over the past 15–20 years [83].

The first mAb-based trial showing overall survival benefit for newly diagnosed advanced TNBC was the IMpassion130 Phase III clinical trial that evaluated the addition of atezolizumab, an anti-programmed cell death protein 1 (PD-1) inhibitor, to standard chemotherapy, resulting in significant improvement in progression-free survival (PFS) and overall survival (OS) for patients with PD-L1 positive tumors, leading the way to immunotherapy for TNBC [84]. Another Phase 3 clinical trial, the KEYNOTE-355, tested pembrolizumab, another PD-1 inhibitor, in patients with advanced TNBC, showing significantly improved overall survival (OS) and progression-free survival (PFS) than chemotherapy alone, in patients whose tumors expressed PD-L1, establishing it as a new standard first-line treatment [85].

In 2019, the US Food and Drug Administration (FDA) granted accelerated approval to the PD-L1 antibody atezolizumab in combination with nab-paclitaxel for advanced or metastatic PD-L1–positive TNBC based on the phase III IMpassion130 trial, followed in 2020 by accelerated approval of the PD-1 antibody pembrolizumab combined with chemotherapy based on the phase III KEYNOTE-355 trial; in 2021, pembrolizumab received full approval, whereas the accelerated approval of atezolizumab was voluntarily withdrawn [86]. The therapeutic effect of PD-1 inhibitors generally correlates with tumor PD-L1 expression, with metastatic TNBC showing a potential response to PD-1/PD-L1 inhibitors [87]. About 40–60% of TNBC patients show PD-L1 expression [88], but only about 40% of these patients benefit from PD-1 inhibitors as monotherapy [89]. Currently, pembrolizumab is the only immunotherapy-based treatment for TNBC, in both advanced and early stages, approved by the US Food and Drug Administration (FDA) in 2020 [85,90].

### 4.3. Antibody–Drug Conjugates (ADC) and Emerging Targets for TNBC Therapies

ADCs are a new class of highly promising therapeutic compounds in TNBC, combining the target specificity of monoclonal antibodies with the cytotoxic potency of chemotherapeutic agents. By selectively binding to tumor-associated antigens that are overexpressed on TNBC cells, ADCs enable the targeted delivery of cytotoxic payloads directly to malignant cells, thereby enhancing antitumor efficacy while limiting systemic toxicity [91]. This approach is particularly relevant in TNBC, where the lack of hormone receptors and HER2 expression restricts the use of conventional targeted therapies. Recent clinical advances have demonstrated that ADCs can achieve meaningful clinical benefit in patients with advanced and metastatic disease, including those with heavily pretreated or chemoresistant tumors [92,93]. Beyond direct tumor cell killing, the robust bystander effect of ADCs may also contribute to functional remodeling of the TME by eliminating antigen-heterogeneous tumor cells and promoting immunogenic cell death, potentially enhancing antitumor immune responses [94,95]. The success of ADCs in TNBC attests their capacity to overcome key limitations of traditional chemotherapy and emphasizes their potential value as a novel targeted treatment in the fight against this aggressive breast cancer subtype.

### 4.4. Emerging Antibody-Based Approaches

Multispecific antibodies are promising new anticancer agents capable of recognizing multiple tumor antigens to eradicate tumor cells with high efficacy [96]. In contrast to natural antibodies, which are monospecific, bispecific or trispecific antibodies recognize two or three different epitopes, respectively, from the same or different antigens [97]. A multitude of strategies have been developed to engineer multispecific antibodies, with more than 100 bispecific antibodies (BsAbs) [98] and 30 trispecific antibodies (TsAb) [99] described so far. Bispecific antibodies and immune engagers, a specific type of multispecific antibody designed to bridge immune cells (like T cells or NK cells) to target cells (like cancer cells) to redirect the immune response, are emerging as innovative strategies to overcome the immunosuppressive TME that characterizes TNBC [100].

By simultaneously targeting a tumor-associated antigen and an immune effector molecule, such as CD3 on T cells, these engineered antibodies facilitate spatially restricted immune synapse formation, promoting direct cytotoxic activity independent of conventional antigen presentation [101]. In TNBC, where heterogeneous antigen expression and limited immune infiltration pose significant therapeutic challenges, bispecific formats offer the potential to redirect endogenous immune cells toward malignant targets while minimizing systemic immune activation [100]. In addition to T-cell engagers, next-generation constructs incorporating dual tumor antigens or immune-modulatory checkpoints, are being developed to enhance specificity and to overcome TME-driven resistance mechanisms [102]. Early preclinical and clinical data suggest that these agents can potentiate antitumor immunity, particularly when integrated with immune checkpoint blockade or other TME-modulating therapies, positioning bispecific antibodies as a promising component of future combination strategies in TNBC [100,103,104].

## 5. Modulation of TME to Enhance mAb Efficacy

### 5.1. Targeting Immunosuppressive Cells

**Figure 2:**
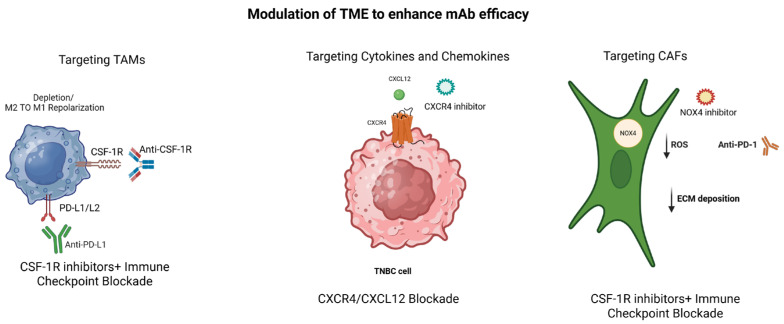
Monoclonal antibody-based therapeutic strategies modulate key cellular and molecular components of the tumor microenvironment in triple-negative breast cancer. Approaches including PD-1/PD-L1 immune checkpoint blockade, antibody–drug conjugates, CSF1R-targeted macrophage modulation, and chemokine axis inhibition (e.g., CXCR4/CXCL12) reshape immune and stromal interactions and may enhance antitumor responses when used in combination regimens. Created in https://BioRender.com.

The therapeutic efficacy of mABs in TNBC is heavily dependent on the immunological and structural context of the tumor microenvironment [105,106]. An immunosuppressive TME, characterized by the abundance of regulatory immune cells, inhibitory cytokines, hypoxia, and dense extracellular matrix, can diminish antibody penetration and effective immune-mediated antitumor responses [105]. Consequently, strategies aimed at modulating the TME have gained increasing attention as a means to enhance mAb efficacy [107]. These approaches include reprogramming or depleting immunosuppressive cell populations, normalizing tumor vasculature, disrupting extracellular matrix components, and targeting cytokine and chemokine signaling pathways that sustain immune exclusion and dysfunction [108]. By reshaping the TME toward a more immune permissive state, these interventions can improve immune cell infiltration, restore effector function, and potentiate the clinical activity of mAb-based therapies [19,109]. Integrating TME modulation with antibody-based treatment therefore represents a rational strategy to overcome resistance and achieve more durable therapeutic responses in TNBC (Figure 2).

### 5.2. Reprogramming Immunosuppressive Cells

Depending on the cellular composition, the immune cells in the TME may differentially influence cancer development and metastasis, with type 1 helper T cells (Th1), cytotoxic T lymphocytes (CTLs), and natural killer cells (NK cells) associated with an immune stimulant microenvironment while the regulatory cells of the TME, including type 2 helper T cells (Th2), TAMs, regulatory T cells (Tregs), and myeloid-derived suppressor cells (MDSCs), associated with an immunosuppressive microenvironment and poor patient outcomes. In addition to immune cells, chemokines and cytokines are critical members of the TME, important to maintain the balance between protumor and antitumor immune responses. The complex crosstalk between cancer cells and TME components affect immunotherapy and other anticancer therapies [19].

### 5.3. Targeting TAMs (CSF1R Inhibitors)

TAMs represent a dominant immunosuppressive cell population within the TME of TNBC playing a prominent role in tumor progression, immune evasion, and resistance to therapy [110,111]. The colony-stimulating factor 1 receptor (CSF1R) signaling axis plays a central role in the recruitment, survival, and polarization of TAMs toward a pro-tumoral M2-like phenotype [112]. Pharmacologic inhibition of CSF1R has therefore emerged as a promising strategy to reduce TAM abundance or reprogram these cells toward a more inflammatory, antitumor state. Preclinical studies have demonstrated that CSF1R blockade can attenuate immunosuppressive signaling, enhance cytotoxic T-cell infiltration, and improve responses to monoclonal antibodies and immune checkpoint inhibitors [113]. Although early clinical trials have shown variable efficacy when CSF1R inhibitors are used as monotherapy, their combination with mAb-based and immune-modulating therapies holds significant potential to overcome TAM-driven resistance mechanisms and improve therapeutic outcomes in TNBC [114].

### 5.4. Depletion or Re-Education Strategies

Beyond direct inhibition of recruitment and survival pathways [115], therapeutic strategies aimed at either depleting TAM or re-educating them toward an antitumor phenotype have gained increasing attention in triple-negative breast cancer [116,117]. Depletion approaches are intended to reduce the overall burden of immunosuppressive macrophages within the TME, thus diminishing macrophage-mediated inhibition of cytotoxic immune responses. In contrast, re-education or reprogramming strategies focus on reshaping macrophage functional states, promoting a shift from M2-like pro-tumoral, anti-inflammatory phenotypes toward antitumoral M1-like phenotypes that support antigen presentation, inflammatory cytokine production, and effective T-cell activation [118]. These interventions can disrupt key mechanisms of immune suppression, enhance antibody-dependent cellular cytotoxicity, and improve responsiveness to monoclonal antibody-based therapies [117]. Although challenges remain in achieving durable macrophage reprogramming without affecting tissue homeostasis, depletion and re-education strategies represent complementary and promising approaches for overcoming macrophage-driven therapeutic resistance in TNBC [118].

## 6. Overcoming Physical and Molecular Barriers

In addition to immunosuppressive cellular components, the TME is characterized also by physical and molecular barriers that limit therapeutic delivery and diminish antitumor immune responses [119]. Dense extracellular matrix deposition, abnormal tumor vasculature, and dysregulated cytokine and chemokine signaling collectively restrict drug penetration, impair immune cell trafficking, and promote resistance to monoclonal antibody-based therapies [120]. Targeting these barriers has therefore emerged as a complementary strategy to improve treatment efficacy [119].

### 6.1. ECM Remodeling and Vascular Normalization

The fibrosis caused by excessive ECM deposition and remodeling contribute to increased tissue stiffness and elevated interstitial pressure within TNBC tumors, creating a physical obstacle to the effective penetration of therapeutic antibodies and immune cells [120,121]. CAFs play a central role in this process by producing collagen, fibronectin, and matrix-remodeling enzymes that reinforce a desmoplastic and immunosuppressive microenvironment [122]. Strategies aimed at modulating ECM composition or enzymatically degrading specific matrix components have shown potential to enhance intratumoral drug distribution and facilitate immune cell infiltration [123]. In parallel, abnormal tumor vasculature, characterized by irregular structure and poor perfusion, exacerbates hypoxia and limits immune surveillance [119]. Vascular normalization approaches seek to restore functional blood flow, reduce hypoxia-driven immunosuppression, and improve the delivery and activity of monoclonal antibodies, thereby creating a more permissive microenvironment for antitumor immunity [124].

### 6.2. Cytokine and Chemokine Blockade

Aberrant cytokine and chemokine signaling within the TNBC tumor microenvironment further reinforces immune exclusion and therapeutic resistance by promoting chronic inflammation and the recruitment of immunosuppressive cell populations [37,39,125]. Elevated levels of pro-tumorigenic cytokines and chemokines contribute to tumor growth, angiogenesis, and immune dysfunction, while simultaneously limiting the effectiveness of immune-mediated therapies. Targeting key cytokine and chemokine axes has therefore gained attention as a means to disrupt these pathogenic signaling networks [70,126]. By inhibiting signals that sustain immunosuppression and stromal activation, cytokine and chemokine blockade can enhance immune cell infiltration, restore effector function, and sensitize tumors to monoclonal antibodies and immune checkpoint inhibitors. Collectively, these strategies underscore the importance of addressing both physical and molecular barriers within the TME to achieve more durable and effective therapeutic responses in TNBC.

## 7. Combination Therapies: mAbs with Chemotherapy or Radiotherapy

Combining monoclonal antibodies with chemotherapy or radiotherapy has become a powerful strategy to improve therapeutic efficacy in TNBC by taking advantage of complementary mechanisms of action [85,127]. Chemotherapy can induce immunogenic cell death, increase tumor antigen release, and modulate immune checkpoint expression, thereby sensitizing tumors to antibody-based therapies, particularly immune checkpoint inhibitors [128,129,130]. In TNBC, the addition of anti-PD-1 or anti-PD-L1 monoclonal antibodies to standard chemotherapeutic regimens has demonstrated improved progression-free and overall survival in selected patient populations, establishing combination therapy as a new standard of care in advanced disease [131,132].

Similarly, radiotherapy has been shown to remodel the TME by increasing antigen presentation, promoting immune cell infiltration, and upregulating immune checkpoint ligands, creating a synergistic platform for mAb activity [80,81,82,133]. Preclinical and early clinical studies suggest that radiotherapy can enhance antitumor immune responses and overcome local immune resistance, supporting its integration with immunotherapeutic approaches in TNBC [134]. Together, these combination strategies intend to maximize antitumor immunity, mitigate resistance mechanisms, and achieve more durable clinical responses than monotherapy alone.

## 8. Personalized Approaches and Biomarker-Guided Therapies

The increasing recognition of the heterogeneity that characterizes TME in TNBC has emphasized the need for personalized therapeutic strategies guided by suitable biomarkers [135,136]. Biomarker-driven approaches aim to identify patients most likely to benefit from monoclonal antibody-based and immune-modulating therapies, while sparing others from ineffective treatments and unnecessary toxicity. In this context, both established and emerging TME-associated biomarkers play a critical role in patient stratification and treatment selection [137,138,139].

### 8.1. PD-L1 Expression and Tumor-Infiltrating Lymphocytes

Programmed death-ligand 1 (PD-L1) expression and tumor-infiltrating lymphocytes (TILs) are among the most clinically validated biomarkers for immunotherapy in TNBC [140]. PD-L1 expression, particularly on immune cells within the TME, has been associated with improved responses to immune checkpoint inhibitors and currently guides the clinical use of anti-PD-1/PD-L1 therapies in advanced and early-stage disease [141,142,143]. Similarly, high levels of stromal TILs correlate with favorable prognosis and enhanced sensitivity to both chemotherapy and immunotherapy, reflecting an active antitumor immune response [144,145]. However, variability in PD-L1 detection assays, spatial heterogeneity of TILs, and dynamic changes in immune infiltration over the course of treatment limit the predictive accuracy of these markers when used in isolation, highlighting the need for integrative biomarker frameworks [146,147].

### 8.2. Emerging TME-Based Biomarkers

Beyond PD-L1 and TILs, a growing number of TME-based biomarkers are being investigated to refine patient selection and predict therapeutic response. These include gene expression signatures associated with immune activation or suppression, markers of myeloid cell infiltration, cytokine and chemokine profiles, and features of extracellular matrix remodeling [148,149,150,151]. Among these, a new genomic test (TNBC-DX) was developed and validated to predict short- and long-term outcomes in patients with early-stage TNBC, concluding that this test was able to predict the pathologic complete response (pCR) and survival outcomes in early-stage TNBC. By enabling more personalized and potentially less intensive treatment approaches, this tool helps align therapeutic strategies with the biological heterogeneity and clinical needs of patients with TNBC [152].

Advances in single-cell sequencing, spatial transcriptomics, and multiplex imaging have enabled high-resolution characterization of the TME, revealing distinct immune and stromal niches that influence treatment outcomes. For example, a comprehensive spatial cell atlas of TNBC generated through an integrated multi-omics approach combining single-cell RNA sequencing and spatial transcriptomics revealed marked heterogeneity in both cellular composition and spatial organization. Normal tissue regions were enriched in functions related to insulin resistance, whereas tumor regions contained diverse cell populations, including immune cells, cancer-associated fibroblasts (CAFs), and mesenchymal cells, underscoring the complexity of the TME and supporting the development of precision oncology strategies [153]. Such technologies provide valuable insights into cellular interactions and functional states within the tumor microenvironment, supporting the identification of novel predictive and prognostic biomarkers relevant to monoclonal antibody and immune-based therapies [149,154,155,156].

### 8.3. Implications for Patient Stratification

The integration of biomarkers based in TME features for clinical decision-making has important implications for patient stratification and the optimization of therapeutic strategies in TNBC [157]. As mentioned early, PD-L1 expression and levels of TILs have been identified as potential biomarkers to predict the response to immune checkpoint inhibitors in advanced TNBC [158,159]. Notably, several FDA-approved diagnostic assays are currently used to evaluate PD-L1 expression in tumor and immune cells, thereby guiding treatment decisions and patient selection for PD-1/PD-L1 immune checkpoint blockade in advanced and metastatic disease [160]. In addition, levels of TILs, a key cellular component of the TME in TNBC, have been associated with prognosis and response to chemotherapy and immunotherapy, supporting their emerging role as clinically relevant biomarkers to guide treatment intensity and therapeutic selection in TNBC [161].

The comprehensive profiling of immune and stromal components facilitates the classification of tumors into biologically distinct subgroups with differential sensitivity to immunotherapy, antibody–drug conjugates, or combination therapies [162]. Patient stratification allows the rational design of adaptive clinical trials and personalized treatment plans that parallels therapeutic interventions with the underlying TME. Ultimately, biomarker-guided personalization has the potential to improve response rates, overcome resistance, and advance precision oncology for patients with TNBC [163].

## 9. Challenges and Future Perspectives

Despite a significant amount of therapeutic approaches targeting the TME in TNBC, many challenges still remain that prevents the broad and long-lasting success expected from antibody-based therapies [14]. Among these challenges, we can mention the intratumoral heterogeneity characteristic of TNBC, which contribute to immune escape by enabling the coexistence of immunologically distinct tumor niches, some of which remain resistant to immune recognition and antibody-mediated cytotoxicity [164,165]. The dynamic adaptation of tumor and stromal cells that involves diverse molecular and cellular mechanisms, such as downregulation of MHC class I, upregulation of immune checkpoint, and immunosuppression driven by myeloid cells, further complicates therapeutic efficacy [165,166,167]. Translational challenges also persist, as promising preclinical findings often fail to fully translate into clinical benefit due to differences between experimental models and human TME, as well as limitations in techniques for biomarker validation and proper patient selection [168,169]. Looking ahead, successful antibody therapies will require integrative strategies that combine advanced spatial and single-cell profiling, novel AI/ML tools for biomarker discovery, innovative trial designs, and rational combination regimens, to simultaneously target the immune, stromal, and vascular components of the TME [170,171]. Such approaches are expected to enhance therapeutic precision, mitigate resistance mechanisms, and ultimately improve long-term outcomes for patients affected with TNBC.

## 10. Conclusions

Therapeutic approaches based on monoclonal antibodies have become important tools with the potential to modulate the TME in TNBC employing mechanisms that target both tumor-intrinsic pathways and several immune and stromal components that influence the progression of the tumor and favor the emergence of therapeutic resistance. Although results obtained from preclinical and clinical studies suggest potential benefits associated with these therapies, the long-lasting effectiveness of these agents may depend on the use of strategies that integrate them into combination regimens that include chemotherapy, radiotherapy, and/or immune-modulating strategies. To have successful treatment outcomes we need to improve patient selection followed by the careful design of approaches that combine personalized regimens based on optimal biomarkers. In this context, novel approaches based on emerging artificial intelligence and machine learning tools may facilitate the integration of complex multiomic and spatial data to support the successful discovery of useful biomarkers, although their clinical value will require further validation in patient cohorts. The application of mechanistic studies and predictive modeling along with robust clinical trials conducted with rigor and guided by suitable biomarkers will be required to develop successful therapeutic interventions. In addition, the validation of predictive markers to define the most effective and long-lasting benefits of these therapeutic strategies against this heterogeneous disease is of the outmost importance.

## Figures and Tables

**Table 1:** Overview of monoclonal antibody-based therapeutic strategies targeting tumor-intrinsic and microenvironmental components, including immune checkpoint inhibitors, TME-targeted monoclonal antibodies, cytokine/chemokine signaling blockade, and bispecific antibodies, in triple-negative breast cancer.

Therapeutic Class	Target	Examples	TME Component Affected	Mechanism of Action	Clinical Status in TNBC *
Immune checkpoint inhibitors	PD-1/PD-L1	Pembrolizumab, Atezolizumab	T cells, tumor cells	Blocks inhibitory signaling to restore T-cell effector function	FDA-approved in selected early and advanced settings
TME-targeted monoclonal antibodies	CSF1R	Cabiralizumab, Emactuzumab	Tumor-associated macrophages	Depletion or reprogramming of immunosuppressive macrophages	Clinical trials
Cytokine/chemokine blockade	CXCR4/CXCL12	Ulocuplumab (anti-CXCR4), Plerixafor **	Immune cells, stromal cells	Disrupts chemokine-mediated immune cell exclusion, tumor–stroma interactions, and metastatic signaling	Preclinical and early clinical studies
Bispecific antibodies	Tumor antigen/CD3	Early TROP-2–CD3 constructs	T cells, tumor cells	Redirects T-cell cytotoxicity toward tumor cells	Preclinical/early clinical

* Clinical status refers to evaluation in breast cancer or solid tumor studies including TNBC, unless otherwise specified. ** Plerixafor is a small-molecule functioning as a CXCR4 antagonist (included here for mechanistic completeness rather than as a monoclonal antibody).

## Data Availability

Not applicable.
